# Computational Analyses Identified Three Diagnostic Biomarkers Associated With Programmed Cell Death for Lung Adenocarcinoma

**DOI:** 10.1155/humu/1743829

**Published:** 2025-08-17

**Authors:** Ting Gong, Bin Jia, Hui Lv, Lili Zeng, Diansheng Zhong

**Affiliations:** ^1^Department of Medical Oncology, Tianjin Medical University General Hospital, Tianjin, China; ^2^Department of Lung Cancer, Tianjin Medical University Cancer Institute & Hospital, National Clinical Research Center for Cancer, Key Laboratory of Cancer Prevention and Therapy, Tianjin's Clinical Research Center for Cancer, Tianjin, China

**Keywords:** biomarker, diagnostic model, immune cell infiltration, lung adenocarcinoma, machine learning, programmed cell death

## Abstract

**Background:** The high morbidity and mortality of lung adenocarcinoma (LUAD) are partly caused by a lack of sensitive and reliable molecular markers for early diagnosis. Programmed cell death (PCD) is a crucial process involved in tumorigenesis and immune regulation, and identifying PCD-correlated genes may contribute to the precision diagnosis and targeted therapy of LUAD.

**Methods:** LUAD samples were acquired from UCSC Xena and Gene Expression Omnibus (GEO) database. PCD-correlated module genes were identified by WGCNA. “Limma” package was employed for screening differentially expressed genes (DEGs) between LUAD and control samples, followed by conducting functional enrichment analysis with “ClusterProfiler” package. Hub genes were selected through machine learning algorithms. Biomarkers for LUAD were screened and further validated by receiver operating characteristic (ROC) analysis. The robustness of the diagnostic model was verified by the confusion matrix. Immune cell infiltration was assessed employing “ESTIMATE” and “GSVA” packages. HALLMARK pathway score was calculated with the “GSVA” package. Transcription factor (TF)–biomarker–chemical network was established using NetworkAnalyst and Cytoscape software. The expressions of the biomarkers in LUAD cells were detected by in vitro experiments. The viability, migration, and invasion of the LUAD cells were measured by CCK-8, wound healing, and Transwell assays.

**Results:** We identified 160 module genes and 5934 DEGs. Then, eight hub genes were selected applying LASSO and support vector machine–recursive feature elimination (SVM-RFE) analyses. Further, *FGR*, *TLR4*, and *NLRC4*, which showed an area under the ROC curve (AUC) > 0.7, were determined as the diagnostic biomarkers for LUAD. Interestingly, they were all low expressed in LUAD samples. We developed a diagnostic model that demonstrated robust performance in distinguishing LUAD samples from normal controls. The three biomarkers showed positive correlation to the infiltration of most immune cells and enrichment in HALLMARK pathways associated with inflammation, immune regulation, and cytokine signaling. Moreover, nine TFs and nine small-molecule compounds targeting the three biomarkers were predicted to construct a TF–biomarker–chemical network. Functional validation revealed that all the three biomarkers were significantly downregulated in LUAD cells. Notably, *FGR* overexpression markedly suppressed LUAD cell proliferation, migration, and invasion.

**Conclusion:** This study identified three PCD-related biomarkers for LUAD diagnosis, providing new potential therapeutic targets.

## 1. Introduction

Lung cancer is an aggressive malignancy and the leading cause of cancer-related mortality worldwide. In 2020, lung cancer accounted for the second highest incidence (11.4% of all cancers) and the highest mortality rate (18.0%) among all cancers [1, 2]. As the most frequent pathological subtype of lung cancer, LUAD has a low 5-year survival rate, posing a major threat to human health [3, 4]. The pathogenesis of LUAD is closely associated with gene mutations, variations of molecular characteristics, and the interactions with tumor microenvironment (TME) [5]. Although recent years have experienced great improvement in the diagnosis and treatments of LUAD, clinical outcomes of LUAD patients still remain unfavorable [6, 7]. At present, immunotherapy can benefit only a small number of LUAD patients, while chemoresistance frequently leads to treatment failure and even disease progression [8]. For cancer patients, tumor recurrence and metastasis are primary causes of poor prognosis and death [9]. Given that early detection could significantly enhance the overall survival (OS) for LUAD patients [10], there is an urgent need to identify specific and sensitive biomarkers for the detection, prognosis, and treatment of LUAD.

Programmed cell death (PCD) is an active and programmed autonomic cell death process that maintains physiological homeostasis and responds to cellular stress in organisms [11]. Currently, at least 12 types of PCD (e.g., apoptosis, ferroptosis, pyroptosis, parthanatos, and necroptosis) with distinct morphological and immunological characteristics have been characterized [12]. PCD involved in the initiation of and progression of tumors is expected to serve as a new target for anticancer therapy in the future [13]. Depending on excretive intracellular compounds, PCD can partially exert antitumor or protumor effects on tumors [14]. A study found that PCD plays a critical part in regulating the initiation and metastasis of LUAD and treatment response [15]. Jia et al. indicated that PCD-related genes are closely linked to the immune cell infiltration, drug sensitivity, and survival of LUAD patients [16]. Additionally, some immune checkpoint inhibitors that target PCD protein-1 or PCD-ligand 1 have been used in advanced LUAD treatment [17]. These findings collectively suggested that elucidating the roles of PCD-related genes may offer new prognostic biomarkers for LUAD patients and help improve the prognosis.

Computational analyses have been widely utilized to discover potential biomarkers for cancers [18]. In the current study, LUAD samples from the UCSC Xena and GEO databases were acquired. Through an integrative approach combining WGCNA and two machine learning algorithms, we determined PCD-related biomarkers for LUAD and used them to develop a diagnosis model to accurately distinguish LUAD samples from control samples. The correlation between immune cell infiltration, HALLMARK pathways, and the biomarkers was analyzed. The transcription factor (TF) and chemicals targeting the biomarkers were predicted to construct a TF–biomarker–chemical network. Finally, the effects of biomarkers on the cell viability, migration, and invasion of LUAD cells were measured by functional assays. Overall, our findings may provide reliable diagnostic biomarkers for LUAD detection.

## 2. Material and Methods

### 2.1. Data Acquisition

The TPM format data of a total of 500 LUAD samples and 397 control samples in The Cancer Genome Atlas (TCGA) database and Genotype Tissue Expression (GTEx) cohort from the UCSC Xena database (https://xenabrowser.net/) were downloaded [19]. The expression data and sample data of LUAD patients in the GSE229705 (123 LUAD samples and 123 control samples) and GSE118370 dataset (6 LUAD samples and 6 control samples) were collected from the GEO database (https://www.ncbi.nlm.nih.gov/geo/) [20]. Here, the samples in TCGA served as the training set, while the GSE229705 and GSE118370 datasets served as external validation sets. Another 1254 PCD-correlated genes were acquired from a previous literature [21].

### 2.2. WGCNA

PCD-related module genes were identified through WGCNA [22]. Specifically, the PCD scores of all LUAD samples and control samples were calculated by ssGSEA using the “GSVA” R package [23, 24]. Using the WGCNA package, we first determined the optimal soft threshold (*β*) through the pickSoftThreshold function to establish a scale-free network topology. Then, based on the topological overlap matrix (TOM), gene coexpression modules (height = 0.25, deepSplit = 2, and minModuleSize = 50) were identified by hierarchical clustering. The crucial module with the highest correlation with PCD score could be identified from the module-trait relationship heat map. The relationship scatter plot for gene significance (GS) for PCD score and module membership (MM) in the critical module was plotted, and the key module genes with GS > 0.2 and |MM| > 0.8 were selected for subsequent analysis.

### 2.3. DEGs and Functional Enrichment Analysis

The DEGs between LUAD samples and control samples in the TCGA and GTEx cohorts were selected by the “limma” R package [25] under the criteria of *p*.adjust < 0.05 and |log2 (fold change)| > 1. The Top 50 DEGs were shown in a heat map. Genes in the intersection of the module genes and DEGs were subjected to comprehensive KEGG and GO enrichment analysis using the “ClusterProfiler” R package [26].

### 2.4. Screening of Hub Genes

The DEGs, module genes, and PCD-related genes were intersected to obtain candidate genes. LASSO regression analysis was performed using the “glmnet” R package [27] (nfolds = 10 and family = “binomial”). The binomial deviance with optimal *λ* was determined by 10-fold cross-validation (CV). Further, SVM-RFE was performed with 10-fold CV to obtain the number of features using the “e1071” R package [28]. Finally, the hub genes were obtained by intersecting the screening results of LASSO and SVM-RFE.

### 2.5. Identification of Biomarkers and Verification

To assess the sensitivity and specificity of hub genes in distinguishing LUAD from control samples, ROC curve analysis was conducted on the TCGA and GTEx cohorts using the “pROC” R package [29]. Meanwhile, the diagnostic values of the hub genes were verified in the GSE118370 and GSE229705 datasets. Genes with the AUC > 0.7 were screened as biomarkers of LUAD and integrated to establish a diagnostic model. Subsequently, the prediction accuracy and robustness of the model were assessed by ROC curve and confusion matrix, respectively [30].

### 2.6. Correlation Analysis Between the Biomarkers and Immune Cell Infiltration

The association between immune cell infiltration and biomarkers was analyzed. Specifically, the ESTIMATE algorithm was utilized to compute the StromalScore, ImmuneScore, and ESTIMATEScore for the biomarkers [31]. The infiltration levels of 28 TILs in LUAD and control samples were calculated by ssGSEA with the “GSVA” R package [32] using the gene set from a previous study [33].

### 2.7. Relationship Analysis Between the Biomarkers and HALLMARK Pathways

The HALLMARK pathway scores were calculated for the biomarkers using the “GSVA” R package [34] using the gene set from the MSigDB (https://www.gsea-msigdb.org/gsea/msigdb/) [35]. Next, the correlation between the biomarkers and HALLMARK pathway scores was analyzed to select pathways with *p* < 0.05 and |cor| > 0.4.

### 2.8. Construction of a TF–Biomarker–Chemical Network

To explore the interactions between TFs, biomarkers, and chemicals, we employed an integrated computational approach. Using the NetworkAnalyst (https://www.networkanalyst.ca/) platform, the TF-gene intersections were predicted using the JASPAR database [36], followed by analyzing the biomarker-chemical relationship via the Comparative Toxicogenomics Database (CTD) [37]. Afterwards, the TF–biomarker–chemical network was developed by the Cytoscape software (Version 3.9.1) [38] under a betweenness threshold > 0.

### 2.9. Cell Lines and Transfection

Dulbecco's modified Eagle's medium (DMEM, OMDCM-036, Oumarsi, China) or F-12K medium (OMD81701, Oumarsi, China) containing 1% penicillin/streptomycin (P/S, Gibco, United States) and 10% fetal bovine serum (FBS, Gibco, United States) was used to culture human lung bronchial epithelial cell line BEAS-2B (VCH00011) and LUAD cell line A549 (VCH00003) acquired from ViCell biotechnology Co., Ltd. (Zhengzhou, China) at 37°C in 95% humidity and 5% CO_2_. Subsequently, the Lipofectamine 3000 (Invitrogen) was utilized to transfect the LUAD cells with pcDNA3.1 vector (V79020, Thermo Fisher) [39] to overexpress *FGR* (oe-FGR). An empty vector served as a negative control (NC).

### 2.10. Quantitative Real-Time PCR (qRT-PCR)

Total RNA of BEAS-2B and A549 cells was first separated using the TRIzol reagent (B610409, Sangon, China) and then reverse-transcribed into cDNA by the RevertAid First Strand cDNA Synthesis Kit (B300538, Sangon, China). Further, qRT-PCR was performed utilizing the SYBR Green Abstract One Step RT-PCR Mix (B110032, Sangon, China). The condition for amplification was as follows: at 95°C for 2 min, 40 cycles at 95°C for 15 s, at 58°C for 30 s, and at 72°C for 30 s. Table S1 lists the primer sequences used. *GAPDH* served as the normalization control for determining relative mRNA expression levels of the biomarkers using the 2^−*ΔΔ*CT^ method [40]. Three biological and technical replicates were performed for each sample.

### 2.11. Cell Viability Detection

The CCK-8 method was employed to detect the effect of *FGR* overexpression on the LUAD cell viability [41]. LUAD cells A549 at a concentration of 5 × 10^4^ cells/well were added into 96-well plates (7512-100-06, AmyJet, China) for 24 h. Next, 10 *μ*L CCK-8 reagent (NDC-RBB-C26QIE-5, AmyJet, China) was filled to each well for another 2 h. Finally, the absorbance value at 450 nm was detected with a multimode reader (SuPerMax 3200, Shanpu, China).

### 2.12. Wound Healing Assay

Wound healing assay was employed to examine the effect of *FGR* overexpression on the migration of LUAD cells [42]. First, the A549 cells (5 × 10^4^ cells/well) were planted into 6-well plates (NRO-AC001-6-10Pack, AmyJet, China). After growing to 100% confluency, the cells were wounded using an aseptic pipette tip and then incubated in serum-free medium for 48 h. The cell images at 0 and 48 h were taken applying an inverted fluorescent microscope (Mateo FL, Leica, Germany). Moreover, ImageJ2 software was utilized to calculate the wound closure (%) of A549 cells.

### 2.13. Transwell Assay

The impact of *FGR* overexpression on the invasion of LUAD cells was evaluated by Transwell assay [43]. First, the diluted Matrigel (RS-2101, Suzhou, China) was precoated on the Transwell chamber (8.0 *μ*m, 3422, Corning, United States). The upper chamber was added with the A549 cells (5 × 10^4^ cells/well) and 150 *μ*L nonserum medium, while the lower chamber was filled with 650 *μ*L complete medium. After culturing for 24 h, the A549 cells invading into the lower chamber were fixed with 4% paraformaldehyde and then colored by 0.1% crystal violet. The invasion pictures were taken with the previous microscope.

### 2.14. Statistical Analysis

All the statistical data were analyzed using R language (Version 3.6.0) and GraphPad Prism 8.0. The Wilcoxon rank test was utilized to examine the difference between two groups of continuous variables. The Pearson method was applied to construct the correlation matrix. Data were represented with mean ± SD, and Student's *t*-test was applied to compare two-group differences. Differences between multiple groups were compared using one-way ANOVA. *p* < 0.05 denoted statistical significance.

## 3. Results

### 3.1. PCD-Related Module Genes in LUAD Were Identified Through WGCNA

**Figure 1:**
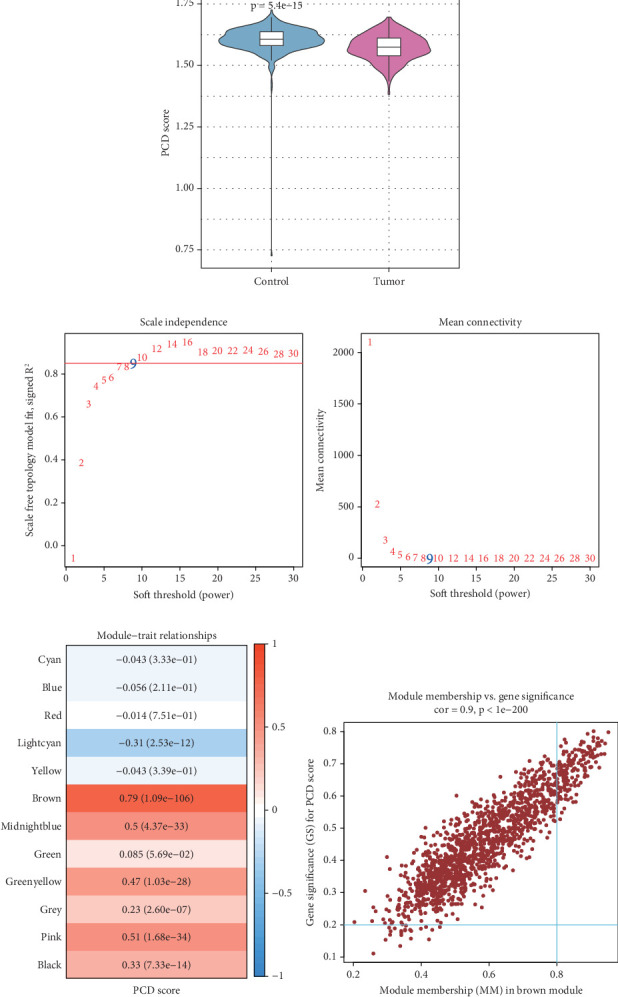
Screening PCD-related module genes in LUAD by WGCNA. (a) PCD score in LUAD samples and control samples. (b) Determining the optimal soft threshold (*β*) to establish a scale-free topology network. (c) Module-trait relationship heat map between each module and PCD score. (d) Scatter plot of module membership (MM) in brown module and gene significance (GS) for PCD score.

The ssGSEA showed that compared to control samples, the LUAD samples had lower PCD scores (Figure 1a). Then, WGCNA was performed to screen critical module genes associated with PCD in LUAD. A scale-free topology network was constructed using an optimal soft threshold (*ꞵ* = 9) (Figure 1b). Hierarchical clustering classified 12 gene coexpression modules, with the brown module showing the strongest positive relation with PCD score (cor = 0.79) (Figure 1c). Further analysis based on MM-GS relationship analysis identified 160 high-confidence genes (GS > 0.2 and |MM| > 0.8) within the brown module for subsequent analysis (Figure 1d).

### 3.2. DEGs and Functional Enrichment Analysis Were Conducted

**Figure 2:**
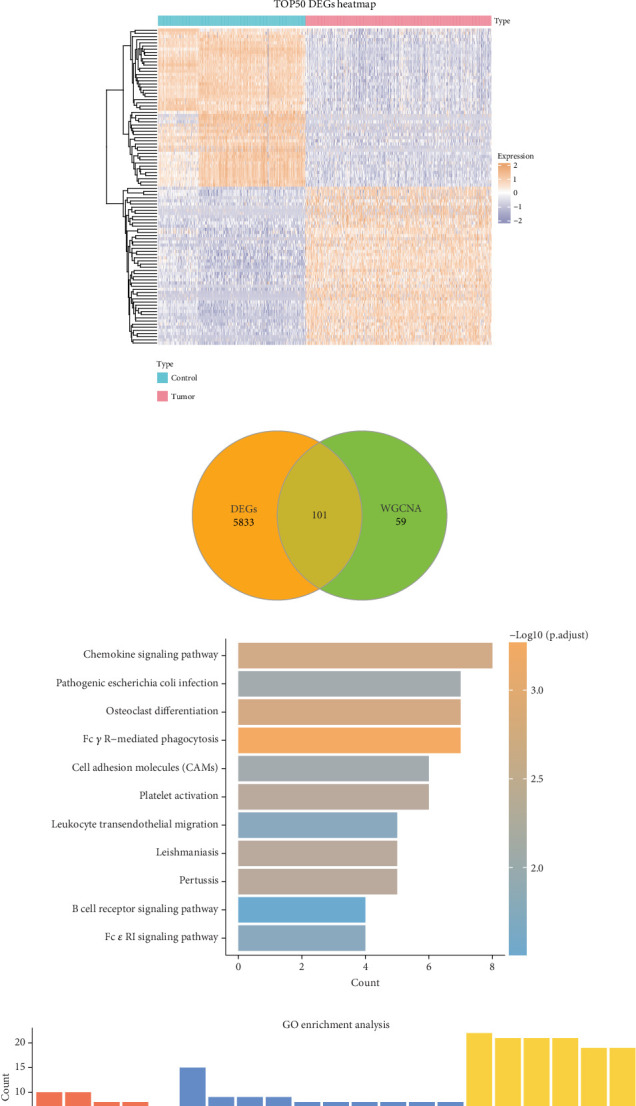
Based on TCGA and GTEx combined dataset to screen for LUAD key differentially expressed genes (DEGs). (a) Volcano plot of DEGs between LUAD samples and control samples in the TCGA and GTEx cohorts. (b) Heat map showing the expression pattern of TOP50DEGs in LUAD and control samples. (c) The Venn diagram shows the intersection of DEGs with PCD module genes screened by WGCNA, and a total of 101 candidate key genes were identified for subsequent analysis. (d) KEGG enrichment analysis of the 101 intersecting genes screened. (e) GO enrichment analysis of the 101 intersecting genes screened.

In the TCGA and GTEx cohorts, a total of 5934 DEGs (3954 downregulated and 1980 upregulated DEGs) were screened between LUAD and control samples (Figure 2a). The expression abundance of Top 50 DEGs was shown in a heat map (Figure 2b). Further, 101 genes in the intersection of the DEGs and module genes were acquired (Figure 2c). KEGG pathway enrichment analysis showed that these common genes were primarily implicated in the chemokine signaling pathway, (CAMs), leukocyte transendothelial migration, Fc gamma R-mediated phagocytosis, Fc epsilon RI signaling pathway, B cell receptor signaling pathway, cell adhesion molecules, etc. (Figure 2d). GO functional enrichment analysis demonstrated that these genes were chiefly enriched in the T cell activation, neutrophil-mediated immunity, neutrophil activation, leukocyte migration, etc., in the biological process term (Figure 2e).

### 3.3. Eight Hub Genes of LUAD Were Screened by Two Machine Learning Algorithms

**Figure 3:**
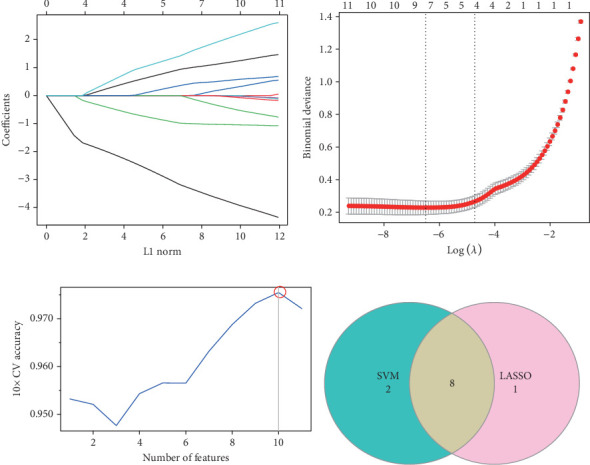
Screening of hub genes for LUAD by two machine learning algorithms. (a) Coefficient variation of gene features in the LASSO regression model. (b) Optimal penalty parameter (*λ*) determined by 10-fold cross-validation (CV). (c) Curve of 10-fold CV accuracy with the number of features in the SVM-RFE model. (d) Venn diagram of SVM-RFE and LASSO results.

First, 11 candidate genes were acquired by intersecting the DEGs, module genes, and PCD-related genes. Then, genes selected by LASSO regression and SVM-RFE analyses were intersected to obtain the hub genes of LUAD. The coefficient of different gene features across different penalty parameters (*λ*) was analyzed by LASSO regression (Figure 3a), and 10-fold CV selected the optimal *λ* as a minimal set of nine genes (Figure 3b). Further, SVM-RFE analysis revealed the highest 10-fold CV accuracy when the feature number was 10 (Figure 3c). Finally, eight hub genes for LUAD were determined by intersecting the results of two machine learning algorithms (Figure 3d).

### 3.4. Three LUAD Biomarkers Were Identified to Construct a Diagnostic Model

ROC analysis of the TCGA and GTEx cohorts revealed strong diagnostic potential of the 11 hub genes, with all AUC values exceeding 0.7 (Figure S1A). These genes showed significantly lower expression in LUAD samples compared to the controls (Figure S1B). The predictive performance and expressions of the 11 genes were consistently validated in GSE118370 and GSE229705 datasets (Figures S1C, S1D, S1E, and S1F). Finally, *FGR*, *TLR4*, and *NLRC4*, which all had an AUC > 0.7 and differential expression across the three datasets, were considered the LUAD biomarkers in this study.

**Figure 4:**
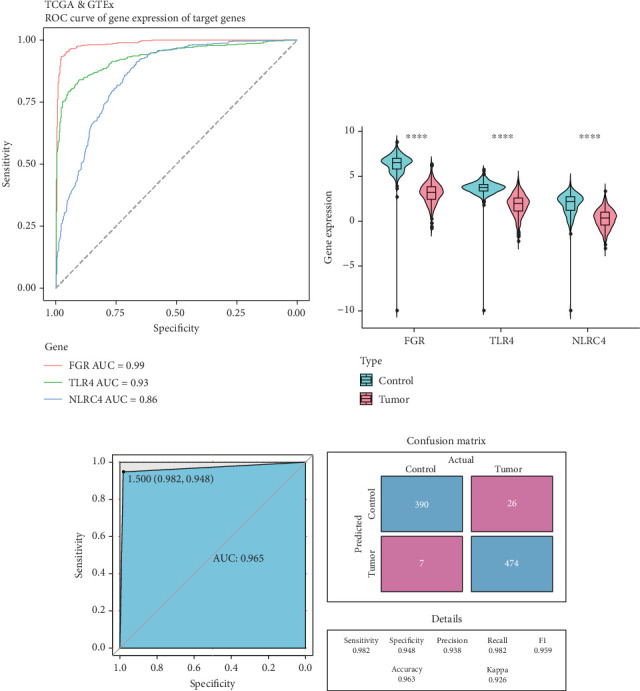
Identification and verification of biomarkers to construct a diagnostic model for LUAD. (a) ROC curves of *FGR*, *TLR4*, and *NLRC4* in the TCGA and GTEx cohorts. (b) Expression levels of *FGR*, *TLR4*, and *NLRC4* in LUAD samples and control samples; ⁣^∗∗∗∗^ means *p* < 0.0001. (c) ROC curve of the diagnostic model. (d) Confusion matrix of the diagnostic model.

The three biomarkers exhibited high specificity and sensitivity in the TCGA and GTEx cohorts, particularly *FGR* (AUC = 0.99), followed by *TLR4* (AUC = 0.93) and *NLRC4* (AUC = 0.86) (Figure 4a). The three biomarkers were all notably low expressed in LUAD samples (Figure 4b). Then, an accurate diagnostic model incorporating the three biomarkers was established, reaching an AUC of 0.965 (Figure 4c). Further, the confusion matrix analysis confirmed the reliability of the diagnostic model, showing high sensitivity (0.982), specificity (0.948), precision (0.938), and overall accuracy (0.963) (Figure 4d). These data suggested the strong robustness of the model.

### 3.5. Correlation Analysis Between Immune Cell Infiltration and the Three Biomarkers

**Figure 5:**
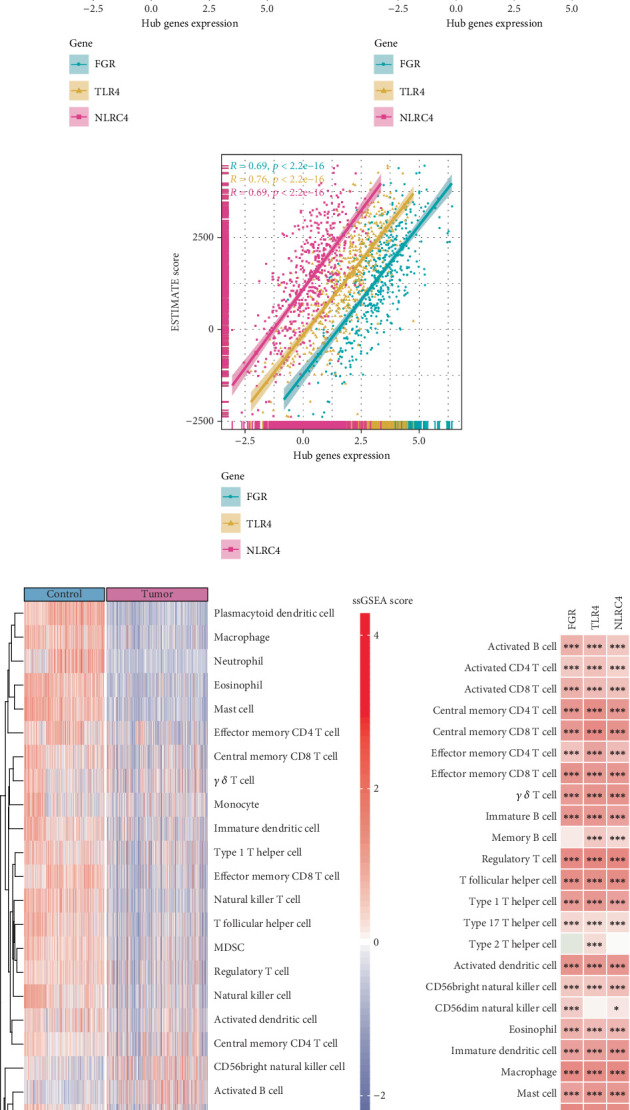
Correlation analysis between immune cell infiltration and the three biomarkers. (a–c) Relationship of StromalScore, ImmuneScore, ESTIMATEScore, and three biomarkers. (d) Infiltration scores of 28 tumor-infiltrating lymphocytes (TILs) in LUAD samples and control samples. (e) Correlation between 3 biomarkers and 28 TILs; ⁣^∗∗∗^ means *p* < 0.001; ⁣^∗∗^ means *p* < 0.01; ⁣^∗^ means *p* < 0.05.

*FGR*, *TLR4*, and *NLRC4* exhibited stronger positive correlation with StromalScore, ImmuneScore, and ESTIMATEScore (*R* > 0.5) (Figures 5a, 5b, and 5c). The ssGSEA analysis of 28 TILs demonstrated markedly lower immune cell infiltration in LUAD samples compared to the controls, particularly for T follicular helper cell, macrophage, neutrophil, mast cell, central memory CD8 T cell, monocyte, immature dendritic cell, natural killer (NK) T cell, effector memory CD4 T cell, and regulatory T cell (Figure 5d). Notably, the expression of *FGR*, *TLR4*, and *NLRC4* was positively linked to most immune cells in LUAD samples, including regulatory T cell, CD8 T cells, mast cell, NK cell, monocyte, T follicular helper cell, B cells, CD4 T cells, and macrophage (Figure 5e). These immune cells may play a crucial regulatory role in the development of LUAD.

### 3.6. The Relationship Between the Three Biomarkers and HALLMARK Pathways

**Figure 6:**
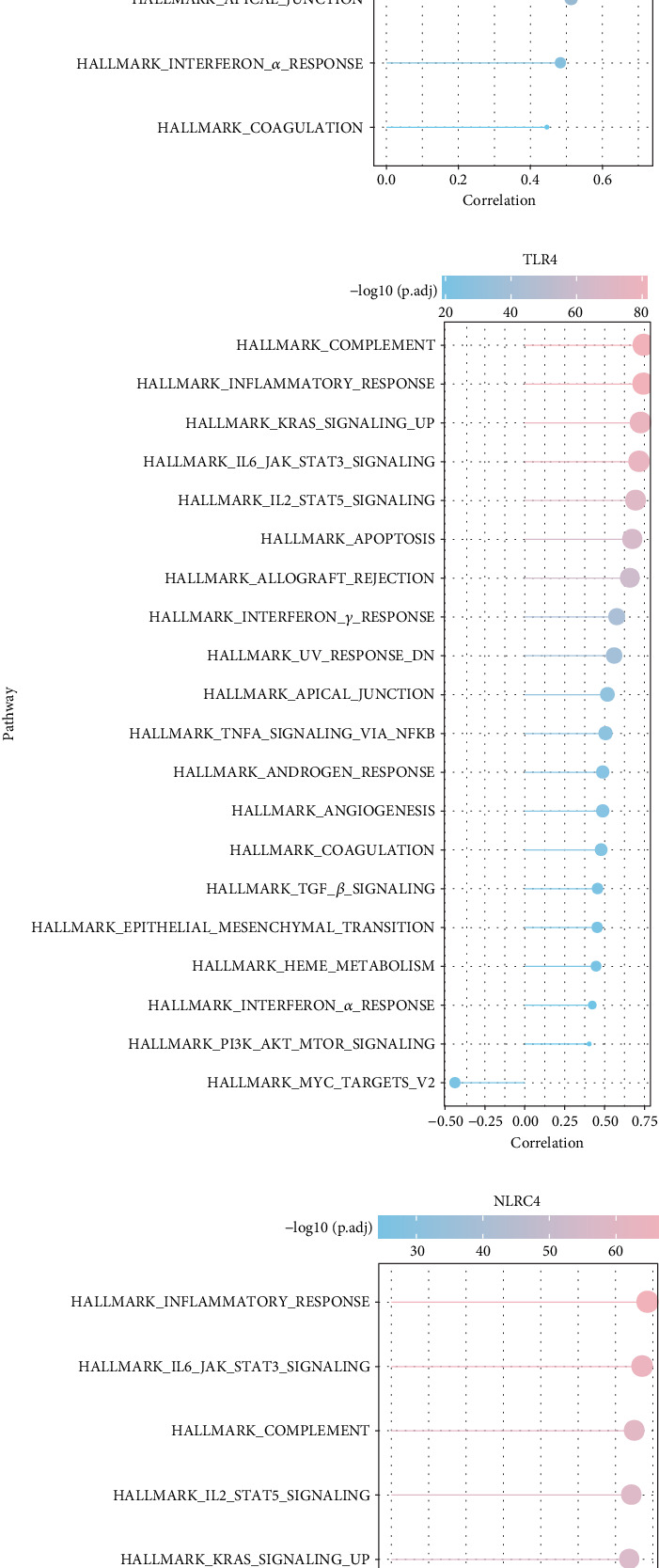
Correlation analysis between HALLMARK pathways and the three biomarkers. (a) Relationship of *FGR* and HALLMARK pathway score. (b) Relationship of *TLR4* and HALLMARK pathway score. (c) Relationship of *NLRC4* and HALLMARK pathway score.

*FGR*, *TLR4*, and *NLRC4* showed a significantly positive relation with many HALLMARK pathways related to inflammation, including IL6 JAK STAT3 signaling, inflammatory response, immune regulation, and cytokine signaling, particularly, IL2 STAT5 signaling, interferon gamma response, and apoptosis (Figures 6a, 6b, and 6c). These findings demonstrated that inflammation and immune regulation–relevant pathways were closely involved in LUAD progression.

### 3.7. TF–Biomarker–Chemical Network Was Constructed

**Figure 7:**
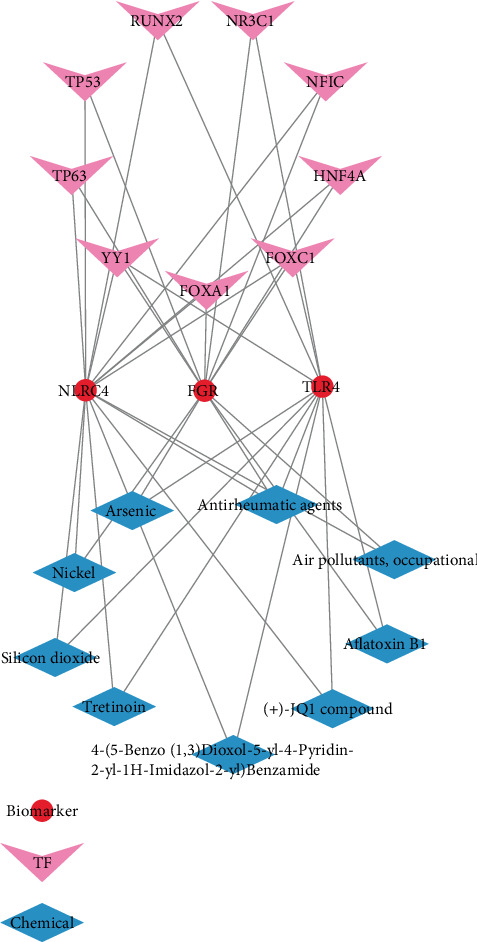
Construction of transcription factor (TF)–biomarker–chemical network.

The TF–biomarker–chemical network was established by Cytoscape software. We identified nine TFs (including *TP53*, *TP63*, *RUNX2*, *NR3C1*, *FOXA1*, *FOXC1*, *HNF4A*, *NFIC*, and *YY1*) potentially regulating the three biomarkers and also nine chemicals (such as arsenic, nickel, silicon dioxide, tretinoin, aflatoxin B1, antirheumatic agents, and air pollutants, occupational) that may influence their expression or function (Figure 7).

### 3.8. Cell Viability, Migration, and Invasion Capabilities of LUAD Cells Were Inhibited by *FGR* Overexpression

**Figure 8:**
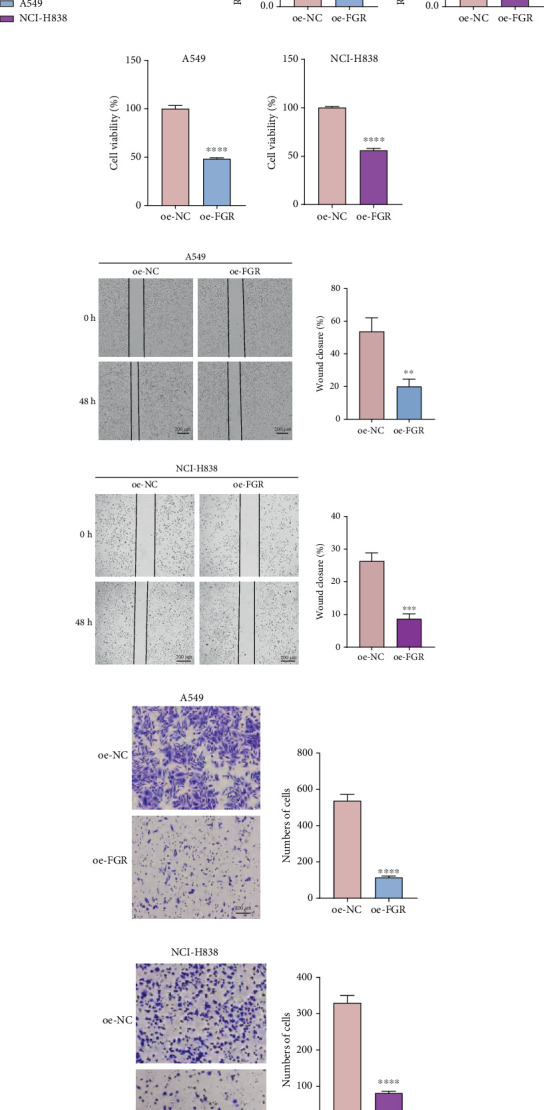
In vitro validation assays utilizing LUAD cells. (a) Relative mRNA expression levels of three biomarkers. (b) qRT-PCR to verify the efficiency of overexpressing *FGR* in A549 and NCI-H838 cells, respectively. (c) CCK-8 assay detecting LUAD cell viability. (d) Wound healing assay assessing the migration capability of LUAD cells. (e) Transwell assay evaluating invasion ability of LUAD cells; ⁣^∗∗∗∗^ means *p* < 0.0001; ⁣^∗∗∗^ means *p* < 0.001; ⁣^∗∗^ means *p* < 0.01. All procedures were three independent repetitive tests.

The qRT-PCR analysis revealed significantly lower mRNA expressions of *FGR*, *TLR4*, and *NLRC4* in LUAD cells (A549 and NCI-H838) compared to human lung bronchial epithelial cells BEAS-2B (Figure 8a). Given the close relation between *FGR* and pathways related to inflammation and immunity [44], we investigated its functional role through overexpression experiments (Figure 8b). Functional assays demonstrated that *FGR* overexpression significantly suppressed the viability (CCK-8 assay, Figure 8c), wound closure (wound healing assay, Figure 8d), and invasion (Figure 8e) of the two LUAD cell lines (A549 and NCI-H838). Additionally, the mRNA expressions of the three key cytokines (IL-6, TNF-*α*, and IFN-*γ*) linked to the inflammation and immune pathway were detected after overexpression of *FGR* (Figure S2A,B). The results showed that *FGR* overexpression significantly downregulated the expression levels of IL-6 and TNF-*α* but significantly upregulated the expression of IFN-*γ*. This change suggested that FGR may play an oncogenic role by suppressing inflammatory responses and activating IFN-*γ*–related antitumor immune pathways in LUAD.

## 4. Discussion

PCD plays critical roles in various biological processes and significantly influences the development of malignancies including LUAD [45]. While previous studies have established PCD-based prognostic models for immunotherapy response evaluation and survival prediction in LUAD patients [46, 47], the early diagnostic potential of key PCD genes remains underexplored. Our study addresses this gap by identifying three stably downregulated PCD-related diagnostic biomarkers (*FGR*, *TLR4*, and *NLRC4*) and developing an effective diagnostic model. Through comprehensive in vitro experiments and immune pathway analyses, we demonstrated the oncogenic effects of these key genes and elucidated their potential mechanisms. These findings may improve the early detection of LUAD, offering substantial clinical translational potential.

FGR encodes Src family tyrosine kinases and functions as a proto-oncogene in cancer development [48]. In LUAD, FGR influences tumor progression by modulating immune cell infiltration and TME responses [49]. Our results showed that the expression of *FGR* in LUAD samples was notably lower compared to the control samples, which was similar to the research of Lu et al. [50]. *TLR4* belongs to the Toll-like receptor family and acts as a critical regulatory factor in innate and acquired immunity [51]. It was reported that *TLR4* is a functional receptor of resistin in lung cancer cell migration and invasion [52]. The expression level of *TLR4* is markedly linked to the histological type, clinical stage, and lymphatic infiltration in non-small cell lung cancer [53]. *NLRC4* is a member of the NOD-like receptor family, and its activation can recruit apoptotic spot proteins and further stimulate Caspase-1, leading to inflammatory response and pyroptosis [54]. *NLRC4* is a critical regulator of the inflammation-related pathway in macrophages and is involved in the growth of CD8 T and CD4 cells, which generate interferon-*γ* [55]. High-expressed *NLRC4* is predictive of a better survival outcome of LUAD patients [56]. We found low-expressed *FGR*, *TLR4*, and *NLRC4* in LUAD samples and used the three genes to develop a diagnostic model (AUC = 0.965) to accurately distinguish LUAD samples from control samples. In vitro assays indicated that *FGR* overexpression could notably suppress the cell viability, migratory, and invasive abilities of LUAD cells. These findings suggested that *FGR*, *TLR4*, and *NLRC4* may be promising diagnostic biomarkers for LUAD.

The TME plays a pivotal role in cancer progression [57]. This study revealed a close relationship between *FGR*, *TLR4*, and *NLRC4* and immune cell infiltration in LUAD samples, including regulatory T cell, CD8 T cells, NK cell, T follicular helper cell, mast cell, monocyte, B cells, CD4 T cells, and macrophage. CD8 T cells are linked to acquired immunity and have a cytotoxic effect on the TME [58]. Macrophage, which normally has a high infiltration level in the TME, fulfills crucial functions in tumor occurrence and metastasis [59]. The accumulation of mast cell in TME is linked to the prognosis and survival in prostate cancer, pancreatic ductal adenocarcinoma, and melanoma [60]. Furthermore, we found that *FGR*, *TLR4*, and *NLRC4* were mainly enriched in the HALLMARK pathways related to immune regulation, inflammation, and cytokine signaling, particularly, inflammatory response, IL2 STAT5 signaling, IL6 JAK STAT3 signaling, apoptosis, and interferon gamma response. Previous clinical and experimental researches have shown that the inflammatory microenvironment facilitates the development of malignancies [61]. The STAT3/STAT5 signaling pathways promote the migration and invasion of tumor cells by enhancing epithelial–mesenchymal transition in LUAD [62, 63]. Interferon gamma is a pivotal cytokine that coordinates tumor-associated immune response, showing the potential of interferon gamma response genes as the risk predictor in LUAD [64]. Therefore, we speculated that *FGR*, *TLR4*, and *NLRC4* may be related to LUAD progression by regulating the immune cell infiltration and inflammatory signaling pathways in TME, which, however, still required further investigation.

There were several limitations to be noted in this study. First, while the training and validation sets derived from the public database provided sufficient data, potential batch effect across different platforms and incomplete clinical features may affect the generalizability of the model. Thus, future validation with multicenter clinical samples incorporating comprehensive patient characteristics (e.g., staging, mutation status, and treatment information) is needed to enhance the clinical adaptability of the model. Second, though we demonstrated a high accuracy of the model in public datasets, the implementation of the model in a clinical setting faces practical challenges including sample processing specifications, uniformity of testing means, and cost-effectiveness. In the future, we will address these issues through prospective multicenter validation and development of noninvasive detection methods. Finally, this study revealed the potential functions of the three biomarkers through immune infiltration analysis, HALLMARK pathway enrichment, and TF–biomarker network analyses; it lacked in-depth mechanistic experiments on the key TFs or downstream signaling pathways. Thus, the follow-up study will focus on the upstream regulators of the key genes and their roles in immune signaling to explore their molecular mechanisms in LUAD.

## 5. Conclusion

To conclude, the current work identified three PCD-related biomarkers (*FGR*, *TLR4*, and *NLRC4*) to construct an effective diagnostic model for distinguishing LUAD samples from normal samples. The three biomarkers were all notably downregulated in LUAD cells, and overexpressed *FGR* could suppress the cell viability, migration, and invasion abilities of LUAD cells. This study offered promising targets for the detection and treatment of LUAD, laying a solid foundation for the research of LUAD pathogenesis.

## Data Availability

The datasets generated and/or analyzed during the current study are available in the GSE229705 repository (https://www.ncbi.nlm.nih.gov/geo/query/acc.cgi?acc=GSE229705) and GSE118370 repository (https://www.ncbi.nlm.nih.gov/geo/query/acc.cgi?acc=GSE118370).

## Nomenclature

AUC: area under the ROC curve
BP: biological process
CAMs: cell adhesion molecules
CC: cellular component
CCK-8: Cell Counting Kit-8
CTD: Comparative Toxicogenomics Database
CV: cross-validation
DEGs: differentially expressed genes
DMEM: Dulbecco's modified Eagle's medium
ESTIMATE: Estimation of STromal and Immune cells in MAlignant Tumor tissues using Expression data
FBS: fetal bovine serum
GEO: Gene Expression Omnibus
GO: Gene Ontology
GS: gene significance
GTEx: Genotype Tissue Expression
KEGG: Kyoto Encyclopedia of Genes and Genomes
LASSO: least absolute shrinkage and selection operator
LUAD: lung adenocarcinoma
MF: molecular function
MM: module membership
MSigDB: Molecular Signatures Database
NC: negative control
PCD: programmed cell death
P/S: penicillin/streptomycin
qRT-PCR: quantitative real-time polymerase chain reaction
ROC: receiver operating characteristic
si: small interfering
ssGSEA: single-sample gene set enrichment analysis
SVM-RFE: support vector machine–recursive feature elimination
TCGA: The Cancer Genome Atlas
TF: transcription factor
TILs: tumor-infiltrating lymphocytes
TME: tumor microenvironment
TOM: topological overlap matrix
TPM: transcripts per million
WGCNA: weighted correlation network analysis

## Supporting Information

Supporting Information Additional supporting information can be found online in the Supporting Information section. Figure S1: Validation of the diagnostic performance of hub genes in LUAD. (A) ROC curves of eight hub genes in the TCGA and GTEx cohorts. (B) Expression levels of eight hub genes in LUAD samples and control samples in the TCGA and GTEx cohorts. (C) ROC curves of eight hub genes in the GSE118370 dataset. (D) Expression levels of eight hub genes in LUAD samples and control samples in the GSE118370 dataset. (E) ROC curves of eight hub genes in the GSE229705 dataset. (F) Expression levels of eight hub genes in LUAD samples and control samples in the GSE229705 dataset; ⁣^∗∗∗∗^ means *p* < 0.0001; ⁣^∗∗^ means *p* < 0.01; ⁣^∗^ means *p* < 0.05; ns means no significant difference. Figure S2: qRT-PCR to verify the effect of overexpression of *FGR* on the expression of inflammatory factors in LUAD cells. (A) Effect of *FGR* overexpression on the levels of inflammatory factors (IL-6, TNF-*α*, and IFN-*γ*) in A549 cells. (B) Effect of *FGR* overexpression on the levels of inflammatory factors (IL-6, TNF-*α*, and IFN-*γ*) in NCI-H838 cells.
