# Supplementary material for: Computational Analyses Identified Three Diagnostic Biomarkers Associated With Programmed Cell Death for Lung Adenocarcinoma
Source: Hum Mutat. 2025 Aug 17;2025:1743829. doi: 10.1155/humu/1743829 (PMC12375862; doi:10.1155/humu/1743829)
Supplement: Supporting Information — Additional supporting information can be found online in the Supporting Information section. Figure S1: Validation of the diagnostic performance of hub genes in LUAD. (A) ROC curves of eight hub genes in the TCGA and GTEx cohorts. (B) Expression levels of eight hub genes in LUAD samples and control samples in the TCGA and GTEx cohorts. (C) ROC curves of eight hub genes in the GSE118370 dataset. (D) Expression levels of eight hub genes in LUAD samples and control samples in the GSE118370 dataset. (E) ROC curves of eight hub genes in the GSE229705 dataset. (F) Expression levels of eight hub genes in LUAD samples and control samples in the GSE229705 dataset; ⁣∗∗∗∗ means p < 0.0001; ⁣∗∗ means p < 0.01; ⁣∗ means p < 0.05; ns means no significant difference. Figure S2: qRT-PCR to verify the effect of overexpression of FGR on the expression of inflammatory factors in LUAD cells. (A) Effect of FGR overexpression on the levels of inflammatory factors (IL-6, TNF-α, and IFN-γ) in A549 cells. (B) Effect of FGR overexpression on the levels of inflammatory factors (IL-6, TNF-α, and IFN-γ) in NCI-H838 cells. [file 1743829.f1.zip › Description of supplementary materials.docx]

**Supplementary figure legends**

**Fig.S1 Validation of the diagnostic performance of hub genes in LUAD.**

(A) ROC curves of 8 hub genes in the TCGA and GTEx cohorts; (B) Expression levels of 8 hub genes in LUAD samples and control samples in the TCGA and GTEx cohorts; (C) ROC curves of 8 hub genes in the GSE118370 dataset; (D) Expression levels of 8 hub genes in LUAD samples and control samples in the GSE118370 dataset; (E) ROC curves of 8 hub genes in the GSE229705 dataset; (F) Expression levels of 8 hub genes in LUAD samples and control samples in the GSE229705 dataset; **** means *p* < 0.0001; ** means *p* <0.01; * means *p* <0.05; ns means no significant difference.
